# Trends in the pursuit of multiple orthopedic surgery fellowships among orthopedic trainees in Saudi Arabia

**DOI:** 10.1186/s13018-022-02928-6

**Published:** 2022-01-24

**Authors:** Abdulaziz Z. Alomar

**Affiliations:** Arthroscopy and Sports Medicine Division, Department of Orthopaedic Surgery, College of Medicine, King Saud University, P.O. Box 7805, Riyadh, 11472 Kingdom of Saudi Arabia

**Keywords:** Orthopedic surgery fellowship, Residents, Fellows, Training

## Abstract

**Background:**

The increase in the enrollment of orthopedic surgery residents in multiple fellowship programs has gained considerable interest. Different factors may determine the specialty and number of fellowships trainees enroll in. This study aimed to elucidate these factors and determinants among orthopedic trainees.

**Methods:**

This is a descriptive cross-sectional study, which was conducted among orthopedic surgical trainees (residency and fellowship training programs) in Saudi Arabia, between March 2021 and May 2021. The data were obtained through an online anonymous questionnaire aiming to clarify the most influential factors that determine the number of fellowships trainees choose, as well as to compare the choice of single fellowships with those of multiple ones.

**Results:**

Two hundred and fifty orthopedic trainees (201 (80%) males and 49 (20%) females) completed the survey. Among them, 216 (86%) and 34 (14%) were residents and fellows, respectively, and 81% (*n* = 203) of the trainees preferred multiple fellowship training, and 22% (*n* = 47) preferred a single fellowship specialty. Notably, the male trainees preferred multiple fellowships to a single one (85% vs. 62%, *p*-value = 0.001), while the female trainees preferred single fellowships to multiple ones (38% vs. 15%, *p*-value = 0.001). The expected rate of income (17% vs. 9%), job opportunities in the private sector (17% vs. 9%), and availability and guarantee of jobs (33% vs. 23%) were the most significant factors that influenced the choices of the participants toward enrolling in multiple fellowships (*p*-values = 0.001, 0.001, and 0.004, respectively).

**Conclusions:**

The study demonstrated that most of the orthopedic trainees in Saudi Arabia prefer the pursuits of multiple fellowship programs. Further, the female trainees preferred single fellowships, whereas the male ones preferred multiple fellowships. The main influencing factors for pursuing multiple fellowships were determined to be private job opportunities, financial gains, and job guarantees.

## Background

Fellowship training programs are an extension of the residency-training journey; they are crucial to preparing the trainees for subsequent practice and careers. However, the pursuit of multiple fellowship specialties in orthopedics has become a recent trend, as demonstrated by the rapid increase in the number of fellowship programs. Moreover, the proportion of orthopedic graduates pursuing fellowship subspecialty training accounts for the increasingly subspecialized practitioners over the past decade [1, 2]. The goal of fellowship training is to produce experts in focused surgical subspecialties and to nurture them into becoming leaders in the given research fields so that they can assume academic roles in training future medical students, residents, and allied health professionals [3]. Fellowships provide additional experience to the trainee and can strengthen a weak area that was not well-addressed during the residency training. Moreover, subspecializing can improve academic and research outcomes within specific orthopedic specialties.

Owing to the increasing number of fellowship-trained surgeons, the modern orthopedic practice trend has shifted from general practice to subspecialized practice in many hospitals, thus decreasing the proportion of general orthopedic surgeons and increasing the number of fellowship-trained surgeons [4]. However, the increase in subspecialized surgeons might cause a shortage of general orthopedists [5] or a lack of interest in the treatment of common orthopedic fractures and emergencies [6]. Moreover, subspecialization improves patient outcomes and care delivery; for example, fellowship-trained surgeons account for a high proportion of performed procedures (78–85%) within their areas of subspecialties [2]. It has been proven that the procedures, which were performed by subspecialized, high-volume surgeons, produced better outcomes than those performed by non-subspecialized ones [7–9].

Presently, the factors influencing the enrollment of residents in multiple fellowship specialties are scarcely explored in the literature; only a few studies have attempted to assess the factors that influence the choices of subspecialties among orthopedic residents [1, 10] or analyze the factors that influence their choices of fellowship programs rather than subspecialties [11]. The existing literature focuses exclusively on trainees who completed single fellowships, and only limited data are available regarding those who pursue multiple fellowships.

This study aimed to determine the factors and motives influencing trainees to pursue multiple fellowships. To the best of the author’s knowledge, this is the first study to elucidate the motives behind the trend of trainees pursuing multiple fellowship subspecialties in the field of orthopedic surgery.

## Materials and methods

This cross section, multicenter study involved orthopedic residents from 44 training centers across Saudi Arabia. The inclusion criteria were as follows: orthopedic residents planning to undergo fellowship training (even those who had not decided on the fellowship specialty) and fellows who had enrolled in the fellowship training program. The exclusion criteria were as follows: residents not planning to undergo fellowship training or those who were unsure. The orthopedic trainees with active email accounts were invited to participate in the online survey via Google Drive. The study was conducted between March 2021 and May 2021.

The 33-item questionnaire comprised components relating to different aspects of fellowship selection and potentially influential factors. The first component of the questionnaire comprised items on demographics, trainees’ opinions regarding the relevance and benefit of pursuing fellowship training, as well as their preferred fellowship specialties. The second component comprised items on influencing and motivating factors that could affect their choices. These influencing and motivating factors were categorized into four items: 1. Experiences and training-related factors; 2. Work-related factors; 3. Specialty characteristic-related factors; and 4. Social factors.

Twenty-one items on the questionnaire were answered based on a five-point Likert-type scale with options ranging from “strongly disagree” to “strongly agree,” and 12 were closed questions with multiple answers. The study was approved by the King Saud University Institutional Review Board (approval date: 18.07.2021/IRB No. 21/0589).

## Statistical methods

The SPSS software, version 23 (SPSS Inc., Chicago, IL, USA), was employed for data entry and statistical analysis. Microsoft Excel was employed for the graphical illustration. Continuous variables were summarized as means and standard deviations (SDs). One-way analysis of variance (ANOVA) was used to determine whether there are any statistically significant differences between the means of two or more independent groups. The categorical variables were summarized as percentages and compared employing the chi-squared or Fisher exact test. Subsequently, a post hoc analysis of the data was performed, employing the adjusted residual values to interpret the in-depth inference of the factors influencing the association between two categorical variables. The survey questions were grouped into the following four factors according to their themes: experience and training-related factors, subspecialty characteristic-related factors, work-related factors, and social factors. Comparative analysis was performed between single and multiple fellowships regarding participants who strongly agreed or agreed on these themed categories. Another comparative analysis was performed between the study groups regarding each influencing factor. All the analyses were performed at a 0.05 significance level.

## Results

### Overall cohort study

In this study, 400 potential participants were reached and included in the survey, although only 265 completed it (response rate = 66%). Fifteen residents were excluded from the study; 6% (*n* = 13) were unsure of their interest in undergoing fellowship programs and 0.9% (*n* = 2) preferred careers in general orthopedic practice to undergoing fellowship. Therefore, the total number of participants after excluding the aforementioned categories of trainees was 250. As already stated, this study included 201 males and 49 females, accounting for 80% and 20% of the population, respectively. Additionally, 216 (86%) and 34 (14%) of the participants were residents and fellows, respectively. The baseline characteristics are shown in Table 1.

**Table 1:** Baseline characteristics for the overall cohort

Characteristic	Overall (*n* = 250)	Single fellowship (*n* = 47)	Multiple fellowship (*n* = 203)	*P*-value
Mean (SD)*				
Age	27 (1.67)	27 (1.78)	28 (1.64)	0.774
Frequency (%)**				
*Gender*				
Male	201 (80)	29 (62)	172 (85)	*0.001*
Female	49 (20)	18 (38)	31 (15.3)	
*Training level*				
Junior	127 (51)	25 (53)	102 (51)	0.801
Senior	89 (36)	17 (36)	72 (36)	
Fellows	34 (14)	5 (11)	29 (14)	
*Marital status*				
Single	148 (59)	30 (64)	118 (58)	0.726
Married	76 (30)	12 (26)	64 (32)	
Married with children	26 (10)	5 (11)	21 (10)	
*Region*				
Central	144 (58)	17 (36)	127 (63)	*0.021*
Eastern	39 (16)	13 (28)	26 (13)	0.082
Northern	3 (1)	0 (0)	3 (2)	0.329
Southern	13 (5)	4 (9)	9 (4)	0.190
Western	51 (20)	13 (28)	38 (19)	0.107

^*^Groups compared using one-way ANOVA

^**^Chi-square test *p*-value comparing single fellowship group to multiple fellowship group

SD: Standard deviation

Junior: Postgraduate years 1, 2, and 3

Senior: Postgraduate years 4 and 5

Most of the trainees strongly agreed that doing a fellowship after residency was necessary to independently practice orthopedics (*n* = 107, 43%, *p*-value = 0.02) and that residency training alone was not sufficient to allow them to practice their desired subspecialties (*n* = 114, 46%. *p*-value = 0.02). Further, 81% (*n* = 203) of the trainees desired multiple fellowships and 19% (*n* = 47) preferred single fellowships. Moreover, 12% (*n* = 30) of the trainees who planned to undergo a fellowship had not yet decided on their preferred specialties. Most trainees had decided their fellowship specialties in their third year of residency (*n* = 72, 29%, *p*-value = 0.04).

A significant number of the study participants believed that trauma and spine surgeries were associated with higher burnout levels compared with other fellowship specialties. Thus, trauma and spine surgeries were selected 195 (78%) and 127 (51%) times, respectively (*p*-value = 0.01).

**Fig. 1:**
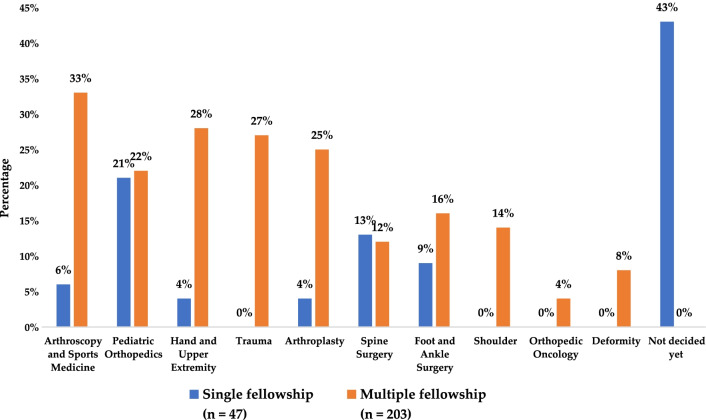
Subspecialty choices for those who opted for single or multiple fellowship

Regarding the participants who opted for multiple fellowships (*n* = 203, 81%), the top three subspecialties that were preferred included arthroscopy and sports medicine (*n* = 67, 33%), hand and upper extremity surgery (*n* = 57, 28%), and arthroplasty (*n* = 51, 25%). Conversely, the three least-selected multiple fellowship subspecialties were orthopedic oncology (*n* = 8, 4%), deformity surgery (*n* = 17, 8%), and spine surgery (*n* = 25, 12%). Regarding the remaining 47 participants who preferred single fellowship, 20 (43%) were undecided about their choices of fellowship subspecialty. However, the three most-selected single fellowship subspecialties were pediatric orthopedics (*n* = 10, 21%), spine surgery (*n* = 6, 13%), and foot and ankle surgery (*n* = 4, 9%) (Fig. 1).

The four most selected multiple fellowship combinations were arthroscopy and sports medicine + hand and upper extremity surgery (*n* = 19, 9%), arthroplasty + trauma surgery (*n* = 17, 8%), arthroscopy and sports medicine + shoulder surgery (*n* = 13, 6%), and arthroplasty + arthroscopy and sports medicine (*n* = 12, 6%) (Table 2).

**Table 2:** Type of combination patterns observed in the multiple fellowship groups

The most common combination categories of fellowship specialties selected by the orthopedic trainees			
Fellowship subspecialties		*N*	(%)
first priority fellowship	second priority fellowship		
Arthroscopy and sports medicine	Hand and upper extremity	19	9.3
Trauma	Arthroplasty	17	8.3
Arthroscopy and sports medicine	Shoulder	13	6.4
Arthroscopy and sports medicine	Arthroplasty	12	5.9
Pediatric orthopedics	Hand and upper extremity	11	5.5
Hand and upper extremity	Shoulder	10	4.9

**Fig. 2:**
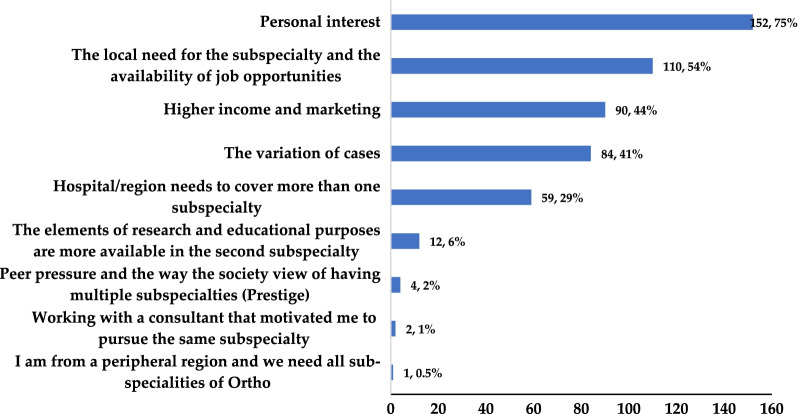
Different reasons the participants gave for selecting multiple fellowships (more than one answered the question) (Percentages calculated from the total number of participants with multiple fellowship (*n* = 203))

Further, regarding the factors that motivated the participants who considered two different fellowships, most focused on personal interests (*n* = 152, 75%), the local necessity for the subspecialty, and the available job opportunities (*n* = 110, 54%) or higher income and marketability (*n* = 90, 44%) (Fig. 1). Finally, more of the participants who opted for multiple fellowships preferred to do so abroad than those who opted for a single fellowship (38% vs. 25%, *p*-value = 0.002) (Fig. 2).

### Factors influencing the choice of fellowship

**Fig. 3:**
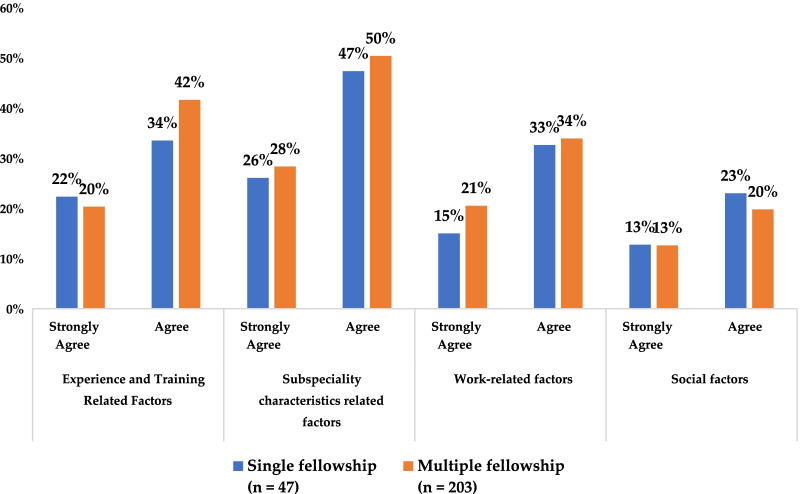
Overall opinions of the single and multiple fellowship groups on the fellowship influencing themed categories

Generally, there were no significant differences between the single and multiple fellowship groups in terms of the four survey-themed categories (Fig. 3), since the participants in both fellowship groups largely agreed, as per the survey-themed categories (Fig. 3). Additionally, personal interest accounted for the highest factor that influenced the choices of the single and multiple fellowship groups, accounting for 49% and 40%, respectively.

### Age of trainees and level of training

There was no statistically significant difference between the mean age of trainees who choose to pursue a single fellowship group versus a multiple fellowships group (*p*-value = 0.774). Additionally, when we compared junior residents (postgraduate year-PGY-1, 2, and 3) to senior residents (PGY 4 and 5) to fellows, there was no statistically significant difference in the percentage of trainees who chose to pursue single or multiple fellowships across different training level groups (*p*-value = 0.801) (Table1).

### Experience and training-related factors

When the single and multiple fellowship groups were compared regarding experience and training-related factors, it was observed that none of the following factors accounted for the choices of multiple or single fellowships: experience during residency training, life experience outside medicine, strengthening area of weakness, and role model/mentor; the two groups were largely similar in these regards (Table 3).

**Table 3:** Comparison of the single and multiple fellowship groups who strongly agreed on similar factors that influenced their fellowship choices

Factors influencing fellowship choice	Single fellowship (*n* = 47)	Multiple fellowship (*n* = 203)	*P*-value*
	*N* (%)	*N* (%)	
*Experience and training related factors*			
Experience during residency training	15 (32)	58 (29)	0.081
Life experience outside medicine	10 (21)	29 (14)	0.062
Strengthening area of weakness	13 (28)	25 (12)	0.066
Role model/mentor	4 (9)	26 (13)	0.062
*Subspeciality characteristic-related factors*			
Surgical skills practiced	16 (34)	57 (28)	0.191
Patients’ volume and variety of cases	11 (23)	37 (18)	0.126
Disease pathology and patient population	10 (21)	49 (24)	0.856
Disease prognosis and surgical outcomes	22 (47)	87 (43)	0.227
*Work-related factors*			
Institution/head of department	4 (9)	19 (9)	0.131
Hospital needs	10 (21)	46 (23)	0.452
Workload, call responsibilities/lifestyle, and work balance	9 (19)	53 (26)	0.088
Expected income rate	4 (9)	35 (17)	**0.001**
Job opportunities in the private sector	4 (9)	35 (17)	**0.001**
Availability and guarantee of jobs	11 (23)	66 (33)	**0.004**
*Social factors*			
Personal interest	23 (49)	81 (40)	0.071
Social and family commitments	4 (9)	19 (9)	0.190
Prestige among the society	2 (4)	14 (7)	0.660
Family and friends’ advice	1 (2)	4 (2)	0.282
Gender-related preferences	1 (2)	9 (4)	0.121

Bold values indicate a p-value less than 0.05 (≤ 0.05) is statistically significant

^*^P value of the chi-square test comparing the strongly agreeing single fellowship participants with the strongly agreeing multiple fellowship participants within each factor

### Specialty characteristic-related factors

The single and multiple fellowship groups were compared regarding the different factors relating to the characteristics of their preferred subspecialties, as well as the influences of these factors on their preferences for multiple fellowships. Specifically, four main factors (surgical skills, disease pathology and patient population, disease prognosis and surgical outcomes, and patients’ volume and case varieties) were considered, and no significant differences were observed between the two groups (Table 3).

### Work-related factors

Comparing the single and multiple fellowship groups in terms of the work-related factors that persuaded the participants to choose multiple fellowships over single ones, it was observed that the expected income rate (17% vs. 9%), job opportunities in the private sector (17% vs. 9%), and availability and guarantee of jobs (33% vs. 23%) were the most significant factors, which the participants strongly agreed on (*p*-values = 0.001, 0.001, and 0.004, respectively) (Table 2). Other factors, such as the request of the institution/head of department, hospital needs, expected workload, call responsibilities/lifestyle, and work balance, were not significantly different between the two groups (Table 3).

### Social-related factors

Overall, most of the trainees strongly agreed that their personal interests were the main drivers of their choices of fellowship (*n* = 104, 42%, *p*-value = 0.01); however, there was no significant difference between the influences of the social factors on the two groups (Table 3). Regarding other factors, such as social and family commitments, prestige, family and friends’ advice, or even their gender, the study participants were largely indecisive, and their opinions were almost evenly distributed between agreeing or disagreeing.

### Gender-based preferences

The difference between the genders opting for single or multiple fellowships was significant. Male participants preferred to pursue multiple fellowships, while female participants preferred to pursue single fellowships (85% vs. 62%, *p*-value = 0.001 and 15% vs. 38%, *p*-value = 0.001, respectively) (Table 1).

## Discussion

The findings here agree with those of a previously reported survey regarding fellowship enrollment; > 94% of the trainees recruited for this study intended to pursue a minimum of one fellowship [1, 2, 12].

Further, 72% of the trainees in this study agreed that the five years of residency training might not be sufficient to prepare them to practice all the orthopedic subspecialties independently; and 77% of this proportion strongly agreed that enrolling in fellowships was necessary to ensure independent orthopedic practice, probably because residency training is aimed at exposing trainees to the basics of most orthopedic subspecialties, as well as prepare them to practice as general orthopedists who can deal with common, simple, and uncomplicated cases in each subspecialty. Furthermore, the expanding body of knowledge, technology, surgical advancements, and subspecialties in orthopedic surgery, as observed over the past two decades, might be a driving force motivating many trainees to pursue additional training before venturing into independent practice, to help them meet the demands of the advancements in orthopedic surgery [13].

An increasing number of practicing orthopedic trainees intend to complete two different fellowships [12, 14] for reasons that are not exactly known. This study is the first to focus on and reveal the main factors influencing trainees to pursue additional fellowship training. The results determined the following as the most significant factors influencing these choices: job guarantee, desired income rate, and increased chances of securing jobs in the private sector. These factors were consistent with the previously reported economic value of undergoing further subspecialty training (studies revealed that fellowship-trained surgeons tend to secure improved job prospects and incomes [4, 15]). Morrell et al. demonstrated the increase in the job opportunities available for fellowship-trained orthopedic surgeons compared with those for non-fellowship-trained ones [15]. Gaskill et al. calculated the estimated return on investment in an additional year of orthopedic training over a working lifetime. They estimated the net present value, internal rate of return, and break-even points. The group observed that adult spine, shoulder and elbow, sports medicine, hand, and adult arthroplasty yield positive returns; trauma surgery yields a neutral return; while pediatrics and foot and ankle surgery exhibited negative net present values [4].

Furthermore, the exploration in this study of the trends in which subspecialty combination patterns were commonly encountered, and it was observed that the most common combination patterns were sports medicine + hand and upper extremity (9%), arthroplasty + trauma (8%), arthroscopy and sports medicine + shoulder (6%), and arthroplasty + arthroscopy and sports medicine (6%). Depasse et al. reported that the three most combined fellowship categories in the US between 2004 and 2016 were arthroplasty and sports, foot and ankle and sports medicine, and sports medicine and trauma [16]. Hariri et al. observed that the most common subspecialty combinations were shoulder/elbow and hand, arthroplasty and sports, and pediatrics and sports [12]. The justifications and incentives behind these choices among trainees are still unclear and can vary; the available literature does not offer evidence of explorations in this area. Thus, to the best of the author’s knowledge, this study is the first to elucidate and explore the underlying factors determining the pursuits of certain combination patterns by those intending to undergo multiple fellowship programs. Comparing the top and bottom three combination patterns, it was observed that the guarantee of job availability was the most significant factor that governed trainees’ choices. Interestingly, sports medicine was the most common fellowship in most combination patterns exhibited in this study and other previously reported ones [12, 16]. Furthermore, this inconsistency between the categories of fellowship combinations among published studies indicates that “related subspecialities” was unlikely to be a primary reason for the combination patterns.

In this study, certain specialties, such as trauma, shoulder, oncology, and deformity, were not selected for single fellowships; the trainees who considered these fellowships intended to do them as combinations with other subspecialties. This is probably because these fellowships increase job guarantees, owing to rarity or the high demand in hospitals for rare specialists, such as trauma, oncology, and deformity specialists, who work in fields where only a few surgeons are available. Although the trauma specialty was perceived by trainees as the specialty associated with the highest burnout level, 27% of the residents still planned to pursue trauma fellowships. However, none of the participants in this study opted for trauma as a single fellowship.

This study was limited by the fact the study results could be affected by the training regulations at where the study has been conducted, and this study only illustrates the situation pertaining to a single country. The organization of orthopedic training varies according to the country [17, 18]. However, according to the training regulations in Saudi Arabia, doing extra training (fellowship) after finishing the residency is not mandatory, and trainees can practice orthopedic independently without pursuing further fellowship training. Furthermore, this study did not investigate the impact of the type of training institutions (university-based vs community-based) or type of hospitals (second level vs third level), or level of trauma service (level-I vs. level III) on the trainee’s fellowship decision. Additionally, the COVID-19 pandemic has recently affected the fellowship and training programs around the world; this could impact fellowship preferences as well [19]. A more extensive analysis would be required to address the same and has not been covered in this study. Another limitation, the results of the study depend on the desire and self-reporting of fellowship selections by trainees who might not necessarily undergo such fellowships in reality or might change their choices and opinions with time. The scope of this study did not include those who planned to undergo more than two fellowships.

## Conclusions

It may be concluded that a large percentage of trainees intend to pursue more than one fellowship. This study is the first to investigate the main factors influencing the pursuits of more than one fellowship and compare that with those of single fellowships. The results revealed that job availability, expected income, marketability, and job opportunities in the private sector were the main influencing factors. This information may assist policymakers and the directors of training centers to analyze the economics of medical education, and ensure the training of orthopedic surgeons in all specialties and subspecialties.

## Data Availability

All data related to this study are available and ready upon request from the author.
